# The I22V and L72S substitutions in West Nile virus prM protein promote enhanced prM/E heterodimerisation and nucleocapsid incorporation

**DOI:** 10.1186/s12985-015-0303-7

**Published:** 2015-05-07

**Authors:** Yin Xiang Setoh, Cindy Si En Tan, Natalie A Prow, Jody Hobson-Peters, Paul R Young, Alexander A Khromykh, Roy A Hall

**Affiliations:** Australian Infectious Diseases Research Centre, School of Chemistry and Molecular Biosciences, The University of Queensland, St Lucia, QLD 4072 Australia; Present Address: Sir Albert Sakzewski Virus Research Centre, Clinical Medical Virology Centre, Herston, QLD Australia; Present Address: QIMR Berghofer Medical Research Institute, Brisbane, QLD Australia

**Keywords:** Flavivirus, West Nile virus, prM, Heterodimerisation

## Abstract

**Background:**

Amino acid substitutions I22V and L72S in the prM protein of West Nile virus Kunjin strain (WNV_KUN_) were previously shown to enhance virus secretion and virulence, but a mechanism by which this occurred was not determined.

**Findings:**

Using pulse-chase experiments followed by co-immunoprecipitation with anti-E antibody, we demonstrated that the I22V and L72S substitutions enhanced prM/E heterodimerization for both the E-glycosylated and E-unglycosylated virus. Furthermore, analysis of secreted particles revealed that I22V and L72S substitutions also enhanced nucleocapsid incorporation.

**Conclusions:**

We have demonstrated mechanistically that improved secretion of virus particles in the presence of I22V and L72S substitutions was contributed by more efficient prM/E heterodimerization.

## Findings

The pre-membrane (prM) and envelope (E) proteins together form the outer structure of West Nile virus (WNV) particles [1,2]. The outer envelope encases a nucleocapsid, consisting of capsid (C) proteins and a single-stranded positive-sensed RNA genome. Incorporation of nucleocapsid into virus particles was proposed to be regulated by coordinated cleavages at the C-prM junction [3]. The prM protein is required to chaperone E [4,5], is essential for viral particle formation [6,7], and prevents premature fusion during virus egress [8]. Mutagenesis studies have led to the discovery of motifs and specific amino acid residues within prM that encode important functions such as virus particle assembly [9-11], prM/E heterodimerisation [12], and virus particle secretion [13,14].

**Figure 1:**
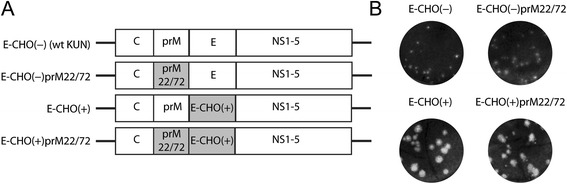
Generation and characterisation of prM22/72 mutant viruses. **A)** Schematics of the viruses constructed for this study, shown with or without E-glycosylation and/or prM22/72 mutations. **B)** Plaque morphology of recombinant viruses were performed on Baby Hamster Kidney cell monolayer, fixed and stained 4 days post infection with crystal violet.

**Figure 2:**
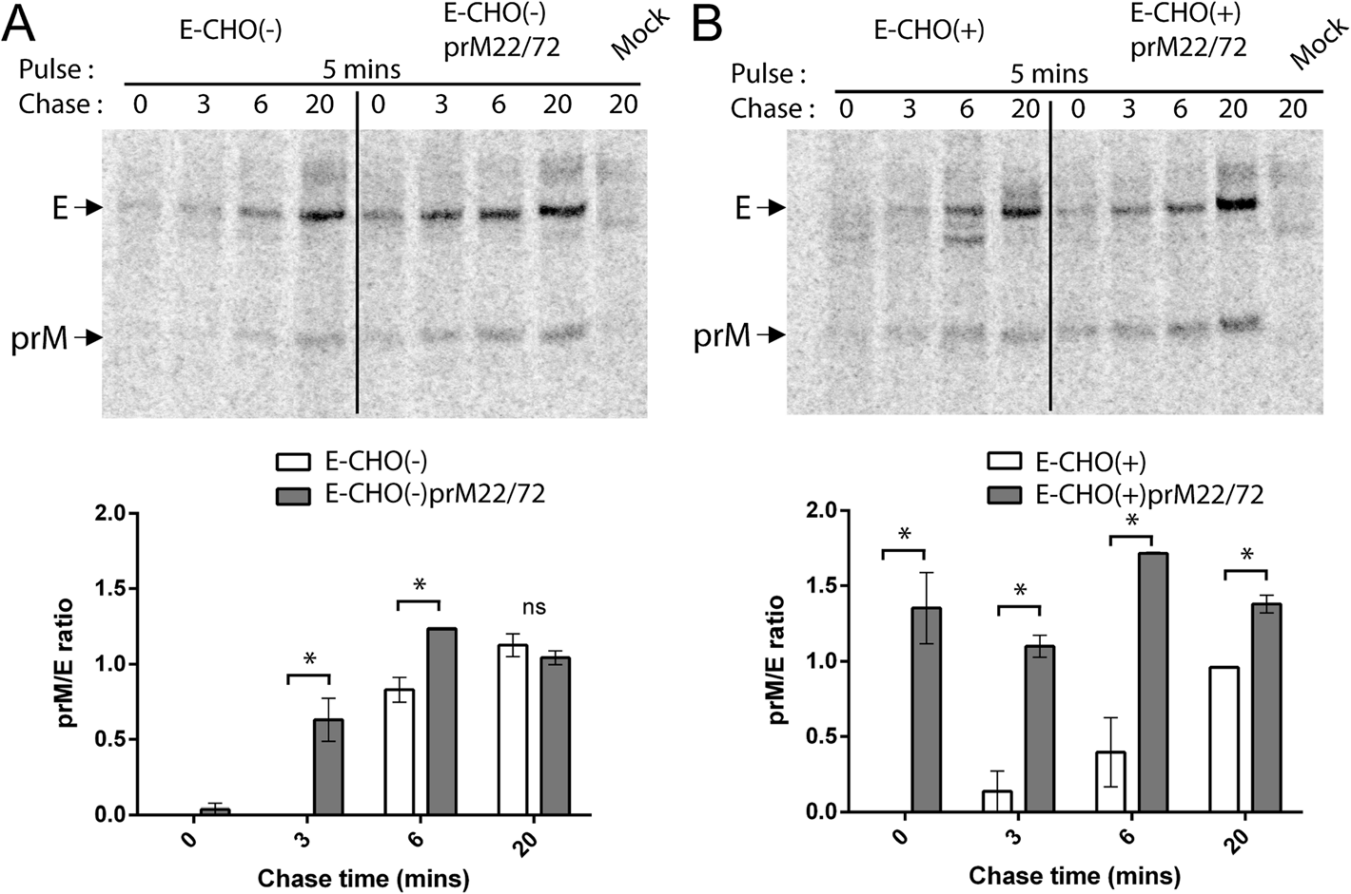
prM22/72 mutations enhanced prM/E heterodimerization. Vero cells were infected with **A)** unglycosylated and **B)** glycosylated viruses at MOI=0.1, and 18 hours later were labelled for 5 mins and chased at 0, 3, 6, and 20 mins. Metabolically-labelled prM was co-precipitated with E using anti-E antibody (3.91D), resolved by SDS-PAGE and exposed on a phosphorscreen. ImageJ analysis was performed to determine the integrated densities of bands and calculated as a prM:E ratio. Bar graphs represent data from two independent experiments. Statistical analysis was performed using *t*-test comparing between samples at each timepoint. * P ≤ 0.05, ns – not significant.

We have previously characterised two amino acid substitutions in Kunjin virus (WNV_KUN_) prM, which when substituted with the corresponding residues of the virulent American New York 1999 strain (WNV_NY99_) (I22V and L72S) resulted in a more stable antigenic structure of the prM protein (analysed using conformational-dependent monoclonal antibody), and promoted efficient secretion of prME and virus particles, as well as increased virulence in weanling mice [15]. In order to identify potential mechanism by which the I22V and L72S prM mutations (prM22/72) exert their effect (s), two previously generated viruses (WNV_KUN_ strain MRM61C, encoding unglycosylated E) with and without the prM22/72 mutations were utilized [15]. Taking into account that E-glycosylation was shown to affect the efficiency of viral particle secretion and virulence [16,17], two additional E-glycosylated mutant viruses (with or without the prM22/72 mutations) were also generated for this study (Figure 1A). The viruses were generated as previously described [15], and passage 2 virus stocks were sequenced to confirm that the introduced mutations were stably maintained. The E-glycosylated viruses formed larger and clearer plaques compared to the E-unglycosylated viruses. Slightly larger plaques were observed for the ECHO (−) prM22/72 (0.92 ± 0.17 mm, n=10) mutant virus compared to the wt ECHO (−) virus (0.52 ± 0.15 mm, n=10) (Figure 1B). This suggested that the enhanced virulence of prM22/72 the mutants were possibly mediated by increased efficiency in virus spread or cytopathic effects in cell culture. We therefore focused on investigating the mechanistic roles of prM protein in the formation of virus particles. Firstly, to characterise prM/E heterodimerisation, Vero cells were infected with respective viruses at MOI=0.1, and at 18 hours post infection cells were starved in methionine/cysteine free media for 30 mins, and then metabolically labelled with ^35^S-methionine/cysteine for 5 mins (pulse). Cell lysates were then collected at chase times of 0, 3, 6 and 20 mins, and co-precipitation of prM was performed using anti-E monoclonal antibody (3.91D) coated onto Protein G Dynabeads (*Invitrogen*). The 3.91D antibody was chosen because it displays strong binding to E protein under reducing conditions on Western blot, indicating that folding of E is not a requirement for binding [18]. Furthermore, it has been mapped to bind in a region in domain III of E (Clarke et al., unpublished results) which is distal to the pr/E interaction sites based on the dengue virus prM/E crystal structure [8]. The amounts of co-precipitated prM and E were measured as corresponding integrated densities determined by ImageJ image processing software (http://imagej.nih.gov/ij/) from two independently conducted experiments, and expressed as prM to E ratio. The prM to E ratio normalizes the amount of prM in relation to E, allowing the accurate quantification of prM/E heterodimerisation efficiency at each time point regardless of E content. prM co-precipitation was detected between 0–3 mins chase for E-CHO (−) prM22/72, and only after 6 mins for E-CHO (−) (Figure 2A). More impressively, prM co-precipitation was already detected at 0 time chase for E-CHO (+) prM22/72, most likely heterodimerising with E during the 5 mins pulse phase, while prM co-precipitation for E-CHO (+) was detected starting from 3 mins chase (Figure 2B). The data indicated that for both E-glycosylated and E-unglycosylated viruses, prM/E heterodimerisation was more rapid with the introduction of the prM I22V and L72S mutations. This could likely be attributed to a more stable prM antigenic structure [15], promoting rapid folding of prM and subsequent heterodimerisation with E protein.

**Figure 3:**
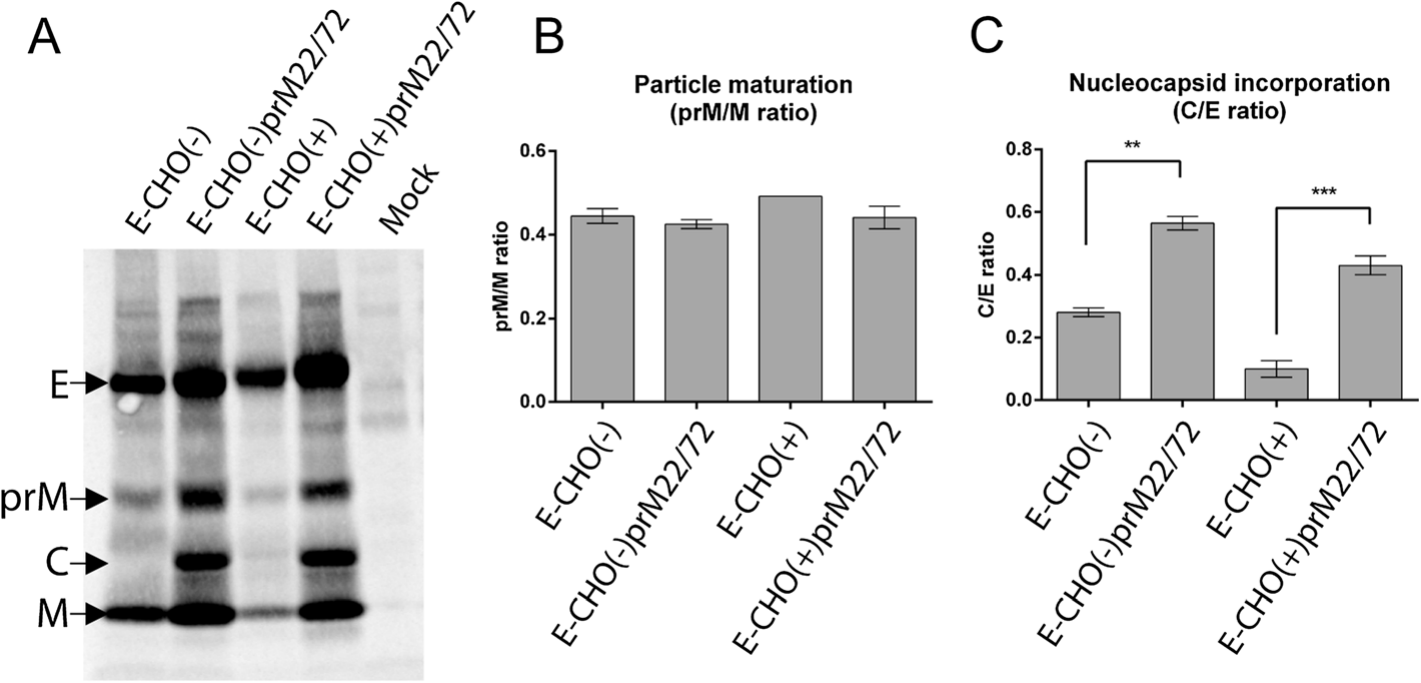
Characterisation of secreted virus particles. **A)** Vero cells were infected at MOI=1 and at 18 hours post infection, were pulsed for 6.5 hours. Culture supernatant was collected and purified by immunoprecipitation using anti-E mAb (3.91D) and resolved on SDS-PAGE. **B)** Ratio of the integrated densities of prM to M bands indicating maturation status of secreted virus particles. **C)** Ratio of the integrated densities of C to E bands indicate the efficiency of nucleocapsid incorporation into virions. Bar graphs represent data from two independent experiments. Statistical analysis was performed by One-way ANOVA with multiple comparisons. ** P ≤ 0.01, *** P ≤ 0.001.

To further investigate how rapid prM/E heterodimerisation affected secretion of virus particles, Vero cells were infected at MOI=1, and at 18 hours post infection, were labelled with ^35^S-methionine/cysteine for 6.5 hours. The metabolically labelled virus particles secreted into the culture media were purified using the 3.91D-coated Protein G Dynabeads. To prevent the disruption of the virus particles, washes were carried out using phosphate buffered saline without Tween-20. The precipitated virus particles were resuspended in NuPAGE LDS Sample Buffer (Novex) and resolved by SDS-PAGE using a NuPAGE 4–12% Bis-Tris Protein Gel (Novex), followed by exposing radiolabelled gel to phosphorscreen (Figure 3A). Integrated densities of the labelled E, prM, C and M bands were measured using ImageJ image processing software from two independently conducted experiments. Firstly, we investigated if the prM22/72 mutations affected furin cleavage of prM (present in immature particles) to M (present in mature particles) by calculating the integrated density ratios of the prM to M bands. No significant differences between the viruses were observed, suggesting that furin cleavage of prM was not affected (Figure 3B). This was expected considering that the prM22/72 mutations lie outside of the predicted furin cleavage site, and have been previously shown not to affect furin cleavage using an *in vitro* furin cleavage assay [15]. The uniform prM to M ratios also served to positively validate our radiolabelled particle assay. Interestingly, the analysis of C to E ratio revealed a significantly increased rate of nucleocapsid incorporation by the prM22/72 mutant viruses compared to wt prM viruses (Figure 3C), suggesting that a more rapidly folding prM protein could potentially incorporate nucleocapsid into virions more efficiently.

## Conclusion

**Figure 4:**
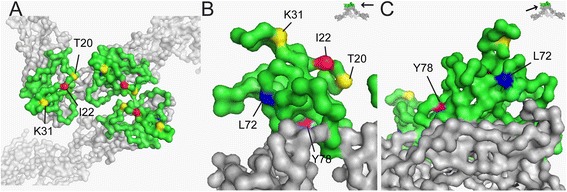
prM residues involved in particle assembly and secretion on the prM/E crystal structure. (PDB: 3C6D, pr in green, E in grey, adapted from Li et al., [8]) **A)** Top view of prME spike showing prM residues I22 (red), T20 and K31 (yellow). **B)** Side view of prM showing I22, T20 and K31 on the same plane position on the top of the prM/E trimeric spike. **C)** Side view showing residues L72 (blue) and Y78 (magenta) facing the prM and E interface.

To date, there have been three separate studies investigating residues in WNV prM involved with particle formation and secretion [11,14,15]. Calvert et al. [14] showed that prM T20 and K31 residues were important for prME particle secretion, with a significant inhibition of prM/E heterodimerisation by the T20D mutation. Although K31 prM mutants did not disrupt prM/E heterodimerisation, an increased accumulation of prME proteins was detected in the endoplasmic reticulum (ER) to Golgi interfaces, demonstrating its role in prME particle secretion [14]. We have previously shown using recombinant V5His-tagged WNV prM, that the I22V mutation enhanced prM export out of the ER [15]. We similarly detected increased presentation of prM on the surface of transfected cells when the I22V mutation was introduced [15]. Interestingly, on the crystal structure of Dengue prM, I22 residue clusters with the T20 and K31 residues, with all three residues sitting on a plane at the apex of the prME trimeric spike (Figures 4A & B). Due to the surface-exposed positions of T20 and K31 residues, it was suggested by Calvert et al. [14], that this region of prM could be involved in protein trafficking (and hence particle secretion) through interaction with host cellular factors [19].

In a study by Tan et al. [11], the Y78 residue in WNV prM was demonstrated to be critical for virus particle assembly. The Y78A mutant produced no particles or virus-induced cellular structures in cells despite readily detectable intracellular E protein. However, prM/E heterodimerisation was not disrupted by the Y78A mutation, which suggests that the defect in the virus particle assembly process could be due to disruption of the higher-order structural re-arrangement of prM/E heterodimers [11]. Notably, we previously reported that a mutation in a nearby residue, L72S, had a significant impact on the antigenic structure of WNV prM [15]. We demonstrated that recognition of L72-prM, by the conformation-dependent monoclonal antibody P10F8, was only successful when co-expressed with E, while S72-prM was structurally stable and recognised by P10F8, independent of co-expression with E [15]. Interestingly, L72 residue is positioned on the same side of prM protein as Y78, and both are found at the interface between prM and E (Figures 4B & C) [11]. Based on these findings, we hypothesize that the region spanning residues 72 to 78 is likely to be important for stabilizing the overall structure of prM in the absence of E, or the higher-order prM/E heterodimeric structures.

In our investigations, we demonstrated that the prM22/72 mutations resulted in enhanced incorporation of capsid protein into secreted virions. This finding was unanticipated as prM and E ectodomains are located on opposite sides of the membrane in relation to the capsid protein. Mutations in the prM ectodomain should therefore not have a direct effect on capsid function. In accordance, the production of smaller prME particles (corresponding to virus-like particles) and larger prME particles (corresponding to virions) in the absence of capsid [20] suggests that prME formation and capsid assembly are independent processes. However, Lobigs et al. [3,21] have elegantly demonstrated that co-ordinated cleavages between C-prM influences the effectiveness of nucleocapsid incorporation into nascent virions, and that delayed signalase cleavage of prM in the ER lumen is critical for nucleocapsid incorporation [22], highlighting the possibility that the I22V and L72S ectodomain substitutions may somehow affect the viral NS2B/NS3 protease and host signalase co-ordinated cleavage events that occur closer to the membrane. Another possible mechanism could be due to the increased affinity of prM for E, and by association of C with prM (in the uncleaved form), this could rapidly draw more capsid molecules into the assembly site, before the C-prM cleavages are completed.

In conclusion, our findings revealed that the I22V and L72S mutations in WNV_KUN_ prM significantly enhanced the heterodimerisation of prM and E proteins. Additionally, we showed that these mutations in prM enhance nucleocapsid incorporation into virions by an as yet unknown mechanism.

## Abbreviations

WNV: West Nile virus
C: Capsid protein
prM: Pre-membrane protein
E: Envelope protein
ECHO+: Glycosylated envelope protein
ECHO-: Unglycosylated envelope protein
